# A Manual of Auscultation and Percussion, Embracing the Physical Diagnosis of Diseases of the Lungs and Heart, and of Thoracic Aneurism

**Published:** 1883-05

**Authors:** 


**A Manual of Auscultation and Percussion, Embracing the Physical Diagnosis of Diseases of the Lungs and Heart, and of Thoracic Aneurism** By AUSTIN FLINT, M. D. Third Edition. Revised. Philadelphia: Henry C. Lea's, Son & Co. 1883.

This work is the product of the author's practical instruction in auscultation and percussion to private classes composed of medical students and practitioners. In the revision for the third edition, the scope of the work originally designed has been restricted to auscultation and percussion, considered chiefly with reference to their practical application, and to present these with as much condensation as possible. We need not assure our readers that Dr. Flint has succeeded in furnishing a manual which the profession has especially prized, and that the present edition, having been carefully revised, is the most valuable work yet published on the important subjects of which it treats.

